# Proteomic meta-study harmonization, mechanotyping and drug repurposing candidate prediction with ProHarMeD

**DOI:** 10.1038/s41540-023-00311-7

**Published:** 2023-10-10

**Authors:** Klaudia Adamowicz, Lis Arend, Andreas Maier, Johannes R. Schmidt, Bernhard Kuster, Olga Tsoy, Olga Zolotareva, Jan Baumbach, Tanja Laske

**Affiliations:** 1 Institute for Computational Systems Biology, University of Hamburg, Hamburg, 22607 Germany; 2 Department of Preclinical Development and Validation, Fraunhofer Institute for Cell Therapy and Immunology IZI, Leipzig, Germany; 3 Chair of Proteomics and Bioanalytics, Technical University of Munich, Freising, Germany; 4 Chair of Experimental Bioinformatics, TUM School of Life Sciences, Technical University of Munich, Freising, Germany; 5 Department of Mathematics and Computer Science, University of Southern Denmark, Odense, 5230 Denmark

**Keywords:** Computational biology and bioinformatics, Systems biology

## Abstract

Proteomics technologies, which include a diverse range of approaches such as mass spectrometry-based, array-based, and others, are key technologies for the identification of biomarkers and disease mechanisms, referred to as mechanotyping. Despite over 15,000 published studies in 2022 alone, leveraging publicly available proteomics data for biomarker identification, mechanotyping and drug target identification is not readily possible. Proteomic data addressing similar biological/biomedical questions are made available by multiple research groups in different locations using different model organisms. Furthermore, not only various organisms are employed but different assay systems, such as in vitro and in vivo systems, are used. Finally, even though proteomics data are deposited in public databases, such as ProteomeXchange, they are provided at different levels of detail. Thus, data integration is hampered by non-harmonized usage of identifiers when reviewing the literature or performing meta-analyses to consolidate existing publications into a joint picture. To address this problem, we present ProHarMeD, a tool for harmonizing and comparing proteomics data gathered in multiple studies and for the extraction of disease mechanisms and putative drug repurposing candidates. It is available as a website, Python library and R package. ProHarMeD facilitates ID and name conversions between protein and gene levels, or organisms via ortholog mapping, and provides detailed logs on the loss and gain of IDs after each step. The web tool further determines IDs shared by different studies, proposes potential disease mechanisms as well as drug repurposing candidates automatically, and visualizes these results interactively. We apply ProHarMeD to a set of four studies on bone regeneration. First, we demonstrate the benefit of ID harmonization which increases the number of shared genes between studies by 50%. Second, we identify a potential disease mechanism, with five corresponding drug targets, and the top 20 putative drug repurposing candidates, of which Fondaparinux, the candidate with the highest score, and multiple others are known to have an impact on bone regeneration. Hence, ProHarMeD allows users to harmonize multi-centric proteomics research data in meta-analyses, evaluates the success of the ID conversions and remappings, and finally, it closes the gaps between proteomics, disease mechanism mining and drug repurposing. It is publicly available at https://apps.cosy.bio/proharmed/.

## Introduction

Technological advancements in proteomics technologies, such as mass spectrometry (MS), have made it possible to study the proteome extensively and on a large scale^1^. The number of articles in proteomics has significantly increased over the past two decades regarding yearly publications from 463 in 2000 to 15,433 in 2022 according to the PubMed database^2^.

The integration of published data is imperative for increasing the sample size and statistical power of own unpublished data. A way to leverage published data is meta-analysis, which is a systematic review of the findings of prior research on a particular topic and combining the results of individual studies. While single studies conducted by the same research group may be influenced by lab-specific biases, meta-analyses can provide a more robust and reliable level of evidence. In fact, meta-analyses are at the top of the evidence hierarchy, which ranks clinical evidence based on its level of independence from different biases that plague medical research^3^. Since meta-analyses can reveal rather global, multi-species biological phenomena, their findings are more likely to be referred to as benchmarks, which is also reflected in the number of citations that are on average higher compared to individual studies^4^.

To facilitate meta-analysis of proteomics data, measurements should be ideally publicly available in raw, unprocessed form. To this end, a measurable set of principles referred to as FAIR data principles, which stands for findable, accessible, interoperable, and reusable, was introduced^5^. Thus, providing raw mass spectra alongside processed data is becoming more important in the proteomics community, making it easier to assess, reanalyze, reuse, compare, and extract new findings from published data. However, many studies are still published with insufficiently annotated raw data or provide only a selection of proteins or genes specified by the authors based on differential expression or other characteristics, such as patient stratification^6,7^. In order to take advantage of published data to search for commonalities and, consequently, potential new sets of biomarkers, which are sets of proteins or genes that may be used to identify a certain pathological or physiological process or disease, the study findings have to be unified to a common ground, i.e., harmonized with respect to the same identifier space and organism.

However, if some studies only provide final lists of biomarker candidates, the evaluation, integration and visualization of biomarkers from different data sets become challenging. There are many approaches to evaluate the discovered set of biomarkers, including pathway enrichment analysis, which reveals biological pathways enriched in a protein list^8^. In silico validation tools like DIGEST can be used to determine the statistical significance of the obtained enrichment scores in contrast to random background models^9^. Additionally, the biomarkers usually only represent a portion of the disease mechanism. Previous research has shown that genes or proteins linked to diseases are not dispersed at random in biological networks. Instead, disease drivers typically reside in structures known as disease modules, which are essentially small subnetworks that represent interconnected mechanisms and can be tied to phenotypic traits^10–13^. In order to determine the underlying disease mechanism and find additional candidate genes or proteins, identified biomarkers can be integrated into a network-based approach for module mining such as ROBUST^11^, DOMINO^12^, or DIAMOnD^13^. The identified disease mechanism can furthermore be searched for known therapeutic targets, i.e., proteins, in these mechanisms that are targeted by registered drugs. The identified drugs can be leveraged as an alternative with cheaper costs and shorter drug development timetables, a process known as drug repurposing^14,15^. According to reports, de novo drug research and development might take 10 to 17 years. Repurposed medications, on the other hand, are often authorized sooner, within 3 to 12 years, and at roughly half the cost^16^.

While the majority of the above tasks can be addressed individually by various tools and websites, a framework that combines all necessary procedures in a user-friendly and interactive manner is missing.

**Fig. 1:**
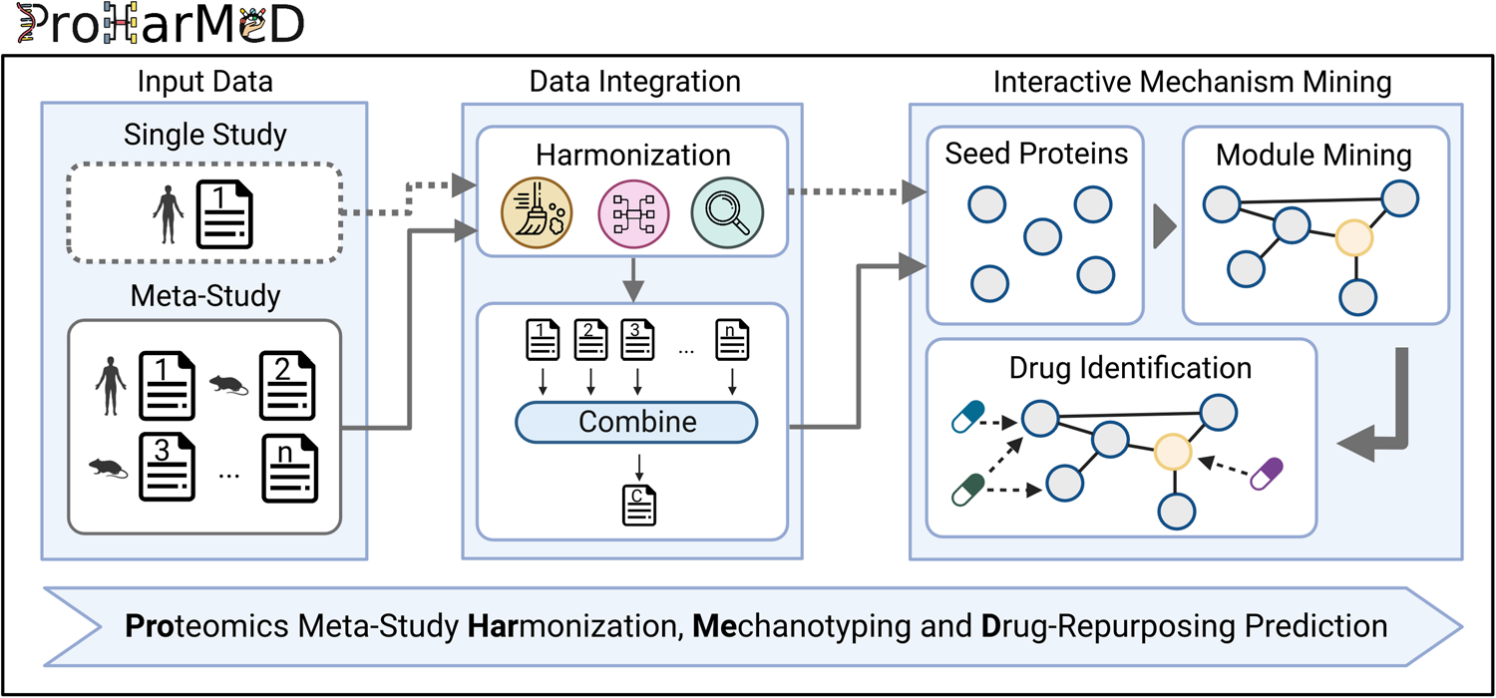
Overview of the pipeline provided by ProHarMeD. The input for ProHarMeD is either one study or a set of studies. Data integration covers steps of filtering IDs, mapping from proteins to genes and finding orthologs of the genes. Finally, integrated biomarker lists can be used as seed nodes for network-based mechanism mining of disease modules which then can be used for drug target and drug repurposing candidate identification. Created with BioRender.com.

Therefore, we provide ProHarMeD (Fig. 1), a web tool to support proteomic data integration by enabling users to harmonize protein and gene IDs of different study data by utilizing existing databases, such as UniProt^17^, MyGene.info^18^, and ID conversion tools, such as g:Profiler^19^. Additionally, it evaluates the success of ID conversion at every step of the remapping procedure. Moreover, the web tool allows for identifying potential biomarkers that may be utilized as seeds for interactive network integration. The user may select from a variety of networks to identify candidate disease mechanisms enriched with seed proteins and examine the resulting candidate mechanisms for potential drug targets and the corresponding drugs. Note, that ProHarMeD supports any tabular user input having a column with either protein IDs or gene IDs, which makes ProHarMeD also suitable for other omics data types, e.g. transcriptomics.

## Results and discussion

### Tool development

**Fig. 2:**
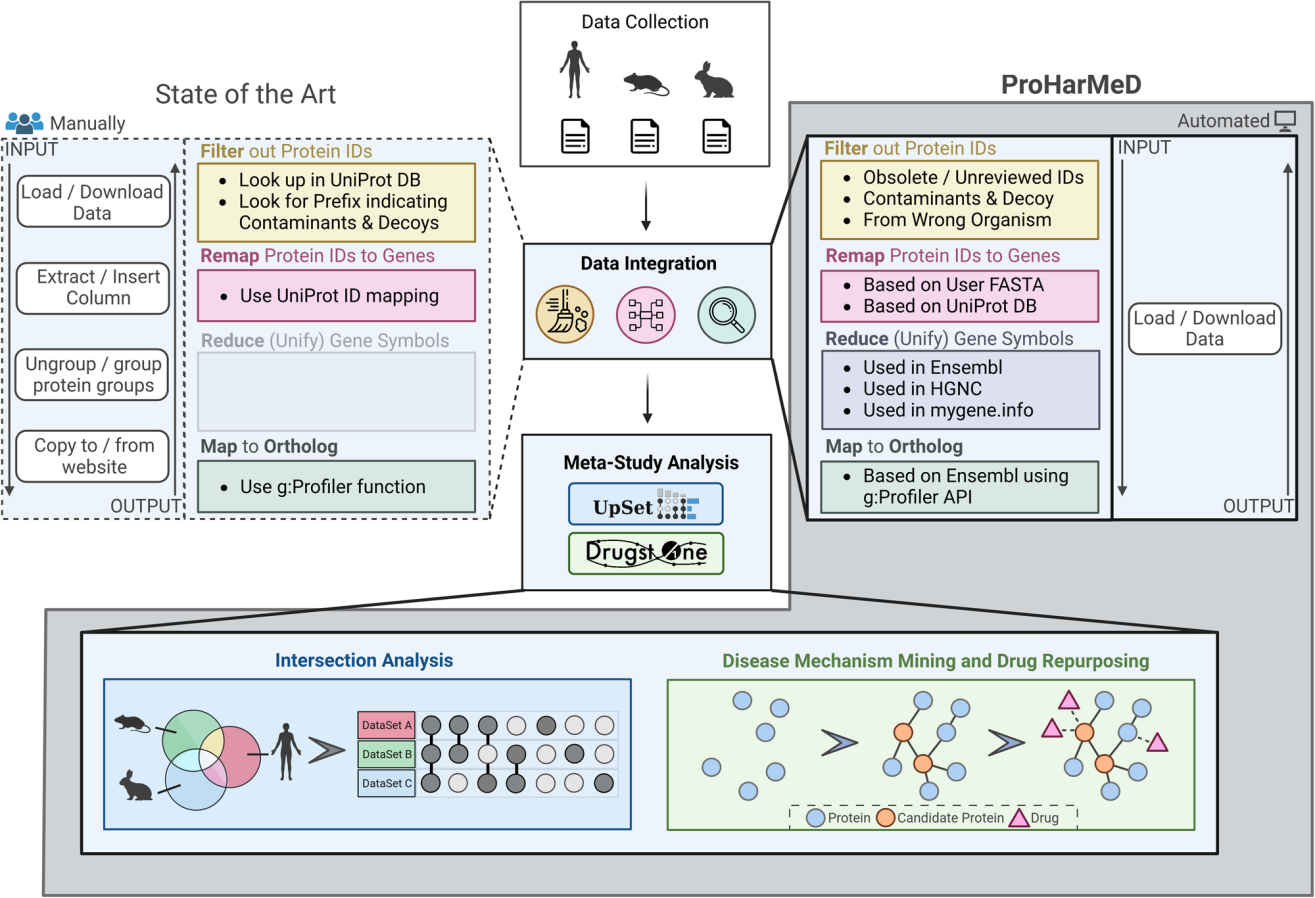
Proteomic data integration, meta-analysis, disease mechanism mining and drug repurposing candidate prediction with ProHarMeD. While currently, many steps in proteomic meta-analyses need to be carried out individually and sequentially by data analysis specialists (left side), ProHarMed offers a streamlined workflow integrated into an easy-to-use web interface closing the gap from multi-study omics data integration via harmonization (right side) to network enrichment and drug candidate extraction (bottom). Created with BioRender.com.

ProHarMeD serves as a comprehensive platform, enabling users to conduct meta-analyses on proteomics data, perform ID conversion and remapping evaluation, and effectively bridge gaps between proteomics, disease mechanism exploration, and drug repurposing. ProHarMeD’s functionality can be divided into three major sections: harmonization, meta-study analysis, and disease mechanisms mining. Data harmonization consists of the following distinct steps: filtering, mapping, and reduction. Meta-analysis comprises an intersection analysis for multi-study biomarker identification, and disease mechanism mining allows for drug target search and identification of repurposable drug candidates (Fig. 2). A detailed description of each step can be found in the method section.

### Use Case 1: Proteomics datasets for meta-analysis

We demonstrate the functionalities of ProHarMeD for proteomics meta-analysis harmonization on four studies investigating osteoblast differentiation and implant-guided bone healing (Table 1). First, the study by Schmidt et al. (2016)^20^ assessed the impact of implant coating compounds such as sulfated glycosaminoglycans on osteogenic differentiation of human bone marrow aspirates. Second, the study by Schmidt et al. (2018)^21^ examined the distinct impact of both a low-sulfated hyaluronic acid derivative and dexamethasone on the osteogenic differentiation of human bone marrow stromal cells in vitro. Third, the study by Calciolari et al. (2017)^22^ examined the protein expression in a Wistar rat calvarial critical size defect model following treatment with scaffold-guided bone regeneration in healthy and osteoporotic conditions and identified up and down-regulated proteins between those two conditions. The combination of mesenchymal stem cells (MSC) and pre-osteoclasts used in bone tissue engineering can repair bone defects more effectively than MSCs alone. Thus, the fourth study by Dong et al. (2020)^23^ assessed the differentially expressed proteins between two treatment groups, either using a combination of MSCs and pre-osteoclasts or MSC-only. Those four studies were performed on different organisms and provided the data in different forms. For two studies, i.e., Schmidt et al. (2016)^20^ and Schmidt et al. (2018)^21^, the raw mass spectrometry data were jointly re-analyzed with MaxQuant^24^. Briefly, mass spectra were matched to protein sequences from UniProt (2021_4, canonical without isoforms). Inferred proteins were organized into protein groups, and only the groups with differential expression were considered. While both Schmidt et al. (2016)^20^ and Schmidt et al. (2018)^21^ datasets provide a list of differentially expressed protein groups, i.e., multiple protein IDs per row, Calciolari et al. (2017)^22^ provides a list of single protein IDs of proteins and Dong et al. (2020)^23^ only provides a list of gene symbols rather than the protein lists from which the authors mapped the gene symbols.

**Table 1:** Overview of the datasets Schmidt et al. (2016)^20^, Schmidt et al. (2018)^21^, Dong et al. (2020)^23^, and Calciolari et al. (2017)^22^ with source organism and data availability.

Study	Organism	Assay	Tissue	MS raw data	No. of protein groups/IDs	No. of gene IDs
Schmidt et al. (2016)^20^	Human	In vitro	EVs	PXD002498	24 groups with 66 IDs	23
Schmidt et al. (2018)^21^	Human	In vitro	EVs	PXD009434	41 groups with 147 IDs	41
Dong et al. (2020)^23^	Mouse	In vitro	ECM	Not provided	Not provided	608
Calciolari et al. (2017)^22^	Rat	In vivo	Bone	Not provided	170 IDs	144

The list of protein groups/IDs and gene IDs is a subset of the raw data which was generated by the authors of the publications by differential expression analysis or other characteristics, such as the suitability to stratify patient groups. The listed studies are performed on either extracellular vesicles (EVs), extracellular matrix (ECM) or bone.

### Fraction of incorrect IDs and redundant gene symbols

**Fig. 3:**
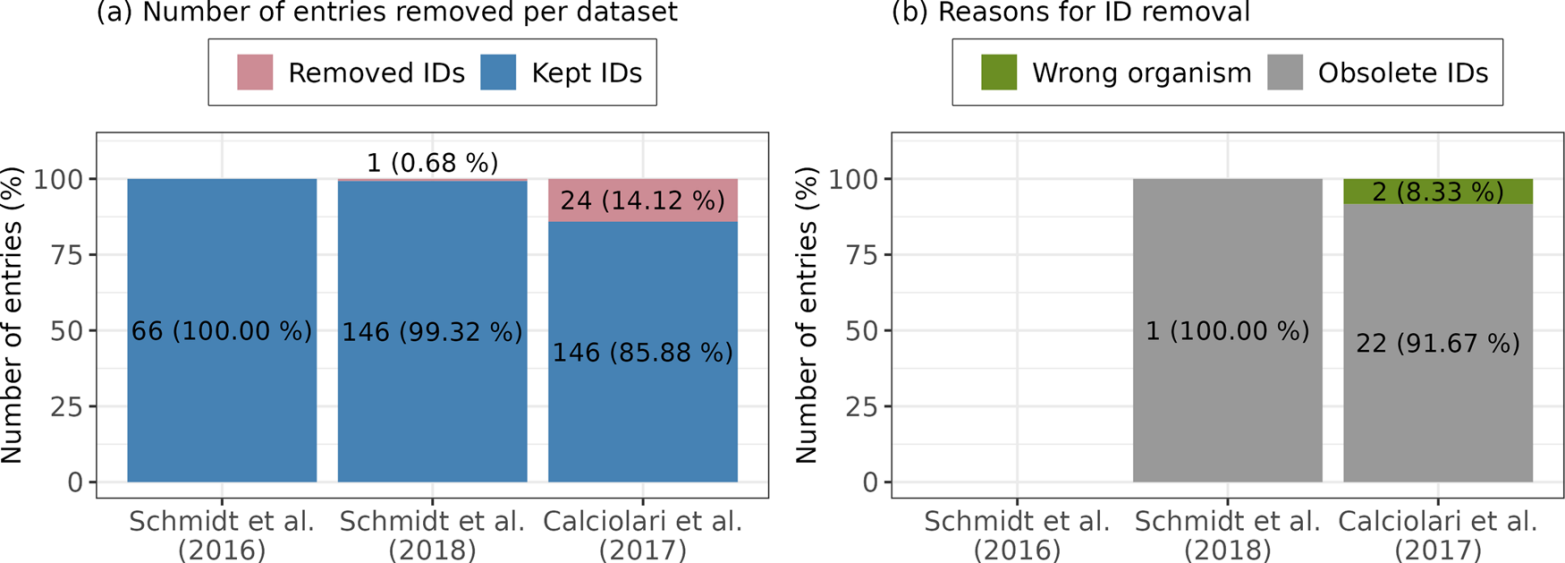
Overview of the “filter_protein_IDs” method results for Schmidt et al. (2016)^20^, Schmidt et al. (2018)^21^, and Calciolari et al. (2017)^22^ datasets. Filtering of Protein IDs cannot be performed on Dong et al. (2020)^23^ data since only Gene IDs are provided. **a** The number of kept and removed IDs for each dataset. **b** Evaluation of reasons that lead to the removal of IDs.

ProHarMeD’s function “filter_protein_ids” reviews protein IDs and removes those of bad quality, i.e., that do not belong to the target organism or are obsolete, which are IDs that were removed in newer UniProt releases. We assessed each protein ID of the protein groups in three datasets with available protein data and filtered out IDs of bad quality (Fig. 3a). Most IDs were removed from the data set Calciolari et al. (2017)^22^. This dataset was generated by Proteome Discoverer which reports one representative protein per protein group. Thus, the removal of an ID leads to the loss of the whole row in the data matrix. The highest fraction of deleted IDs was obsolete (Fig. 3b). Interestingly, in the dataset from Calciolari et al. (2017)^22^ the two IDs *B4DQ80* and *B7Z722* were removed since they were assigned to the wrong organism, i.e., both IDs are from human, while the study was performed in rats. A reason for the occurrence might be the high similarity of *B4DQ80* to rat gene tropomyosin 3gamma (*Tpm3*), which is encoded by the rat protein *Q63610* and also present in the published protein list. Therefore, we assume that those IDs are not relevant hits due to being from the wrong organism and filter out these IDs. The ID removal is tracked in ProHarMeD’s log files which allow the user to assess if the removal was appropriate.

### Gain of enrichment terms related to the reduction of newly mapped gene names

**Fig. 4:**
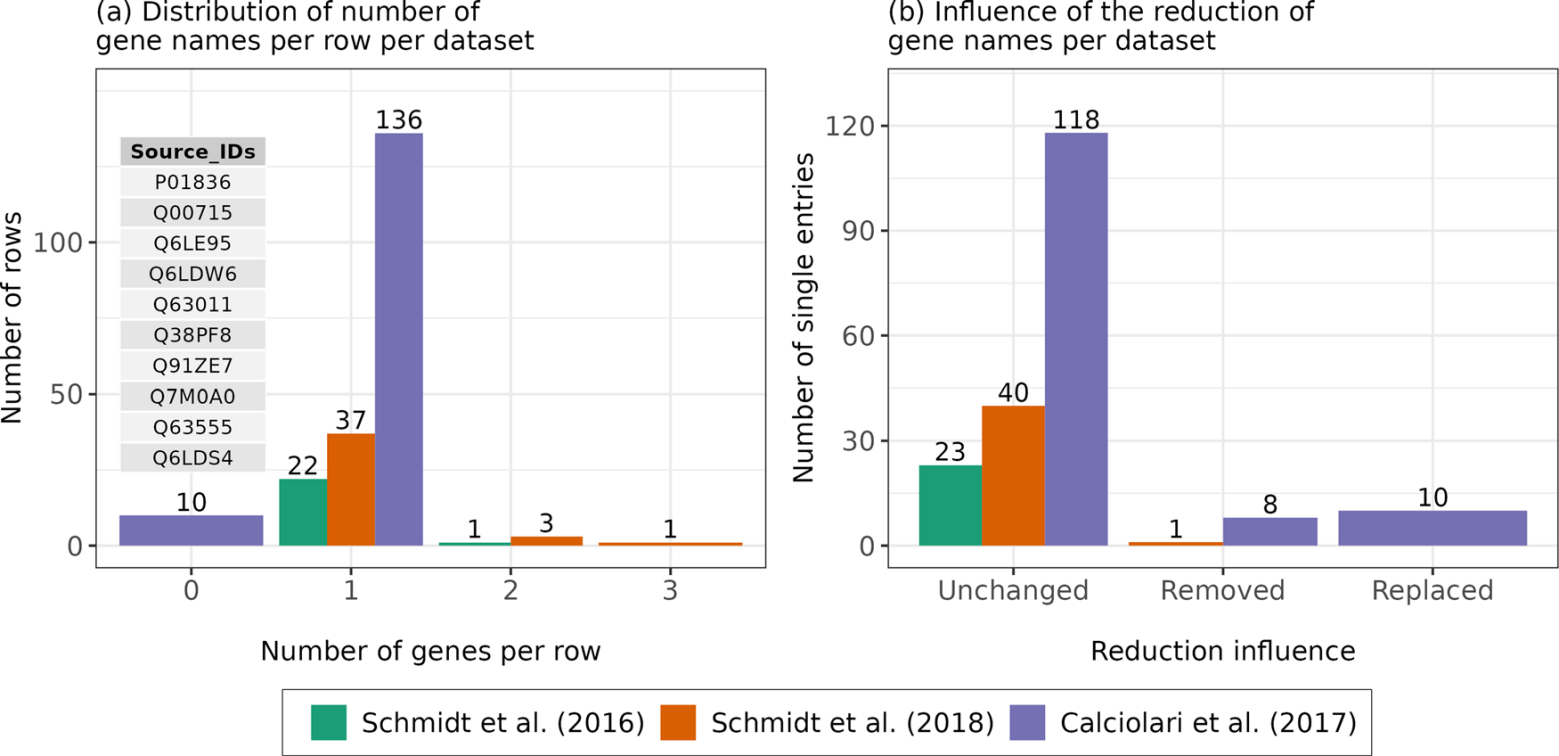
Mapping and reduction to common identifier space of gene names. **a** Distribution of the number of gene names mapped from protein groups per dataset. Ten protein IDs shown in the table as source IDs (first column) do not have an official gene name in UniProt and are, therefore, filtered out. **b** Reduction of the resulting mapped gene names, upon removal of the 10 protein IDs from (**a**), based on mappability in Ensembl ID space.

Since the dataset by Dong et al. (2020)^23^ only provides a list of gene symbols, which we refer to as gene names here, it is necessary to translate the protein IDs from studies Schmidt et al. (2016)^20^, Schmidt et al. (2018)^21^, and Calciolari et al. (2017)^22^ into the same gene annotation. To accomplish this, ProHarMeD’s “remap_gene_names” function uses the UniProt API linking the protein IDs to the primary gene names included in the UniProtKB. Calciolari et al. (2017)^22^ dataset contains a list of individual protein IDs, whereas datasets from Schmidt et al. (2016)^20^ and Schmidt et al. (2018)^21^ contain protein groups discovered by the previously described MaxQuant re-analysis of raw MS data (Section Proteomics datasets for meta-analysis). As a result, datasets from Schmidt et al. (2016)^20^ and Schmidt et al. (2018)^21^ contain several gene names assigned to each row (Fig. 4a). Additionally, 10 protein IDs from the Calciolari et al. (2017)^22^ dataset are missing gene name annotations based on UniProt. Although the user can choose to keep the rows with such IDs in the dataset by setting a checkmark on “keep empty”, allowing to search for missing names manually, these rows are eliminated here for simplicity. Consequently, we only rely on fully annotated protein IDs by UniProt. Redundancy after remapping to gene names from a protein group inside a single row may occur because a particular gene may have more than one name. The fact that different databases use varying gene names as primary identifiers is an additional issue. This can be tackled by ProHarMeD’s “reduce_gene_names”, for instance, on the ground of Ensembl IDs^25^ (Fig. 4b), or other grounds listed under the method section “Reduction of gene names.”

**Fig. 5:**
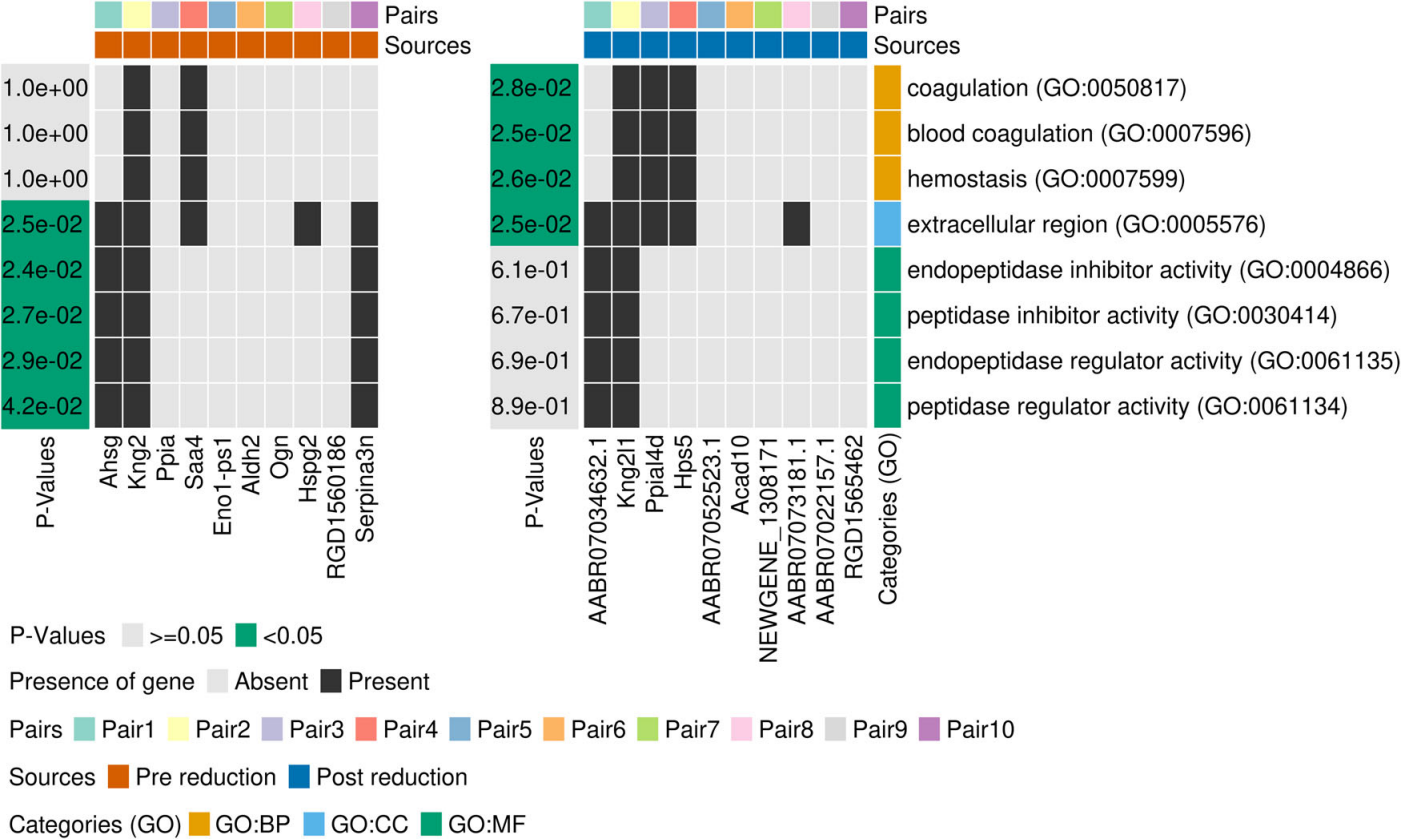
Enrichment results for the replaced 10 genes from Calciolari et al. (2017)^22^ (Fig. 3b), ran with the set before and the set after reduction by applying the method “reduce_gene_names” colored by the set source, respectively. The significantly annotated enrichment terms (FDR < 0.05) are sorted by the Gene Ontology^67^ categories cell component (CC), molecular function (MF) and biological process (BP) and colored by their *p*-value for each of the 2 gene sets. For each gene, black denotes inclusion in the intersection for the enrichment term and gray denotes exclusion. Finally, to indicate each pre and post-reduction candidate combination, the groups are summarized into pairs and colored accordingly.

In order to assess the influence of the reduction step the 10 gene names of study Calciolari et al. (2017)^22^ that were replaced by their annotated gene name in Ensembl (Fig. 3b, third column) have been further inspected by comparing the original gene names to their replacements (Fig. 5). For that we ran an enrichment analysis with g:Profiler^19^ on the set before reduction and after the reduction separately and compared the significantly enriched annotation terms. Noticeably, new associations were determined as significant, particularly those related to biological processes, while associations related to molecular functions became insignificant. The reason for that is the two genes *Ppia* (reduced: *Ppial4d*) and *Serpina3n* (reduced: *RGD1565462*) for which either only the pre-reduction or post-reduction gene name has annotations according to g:Profiler.

### Comparing biomarkers of different organisms

**Fig. 6:**
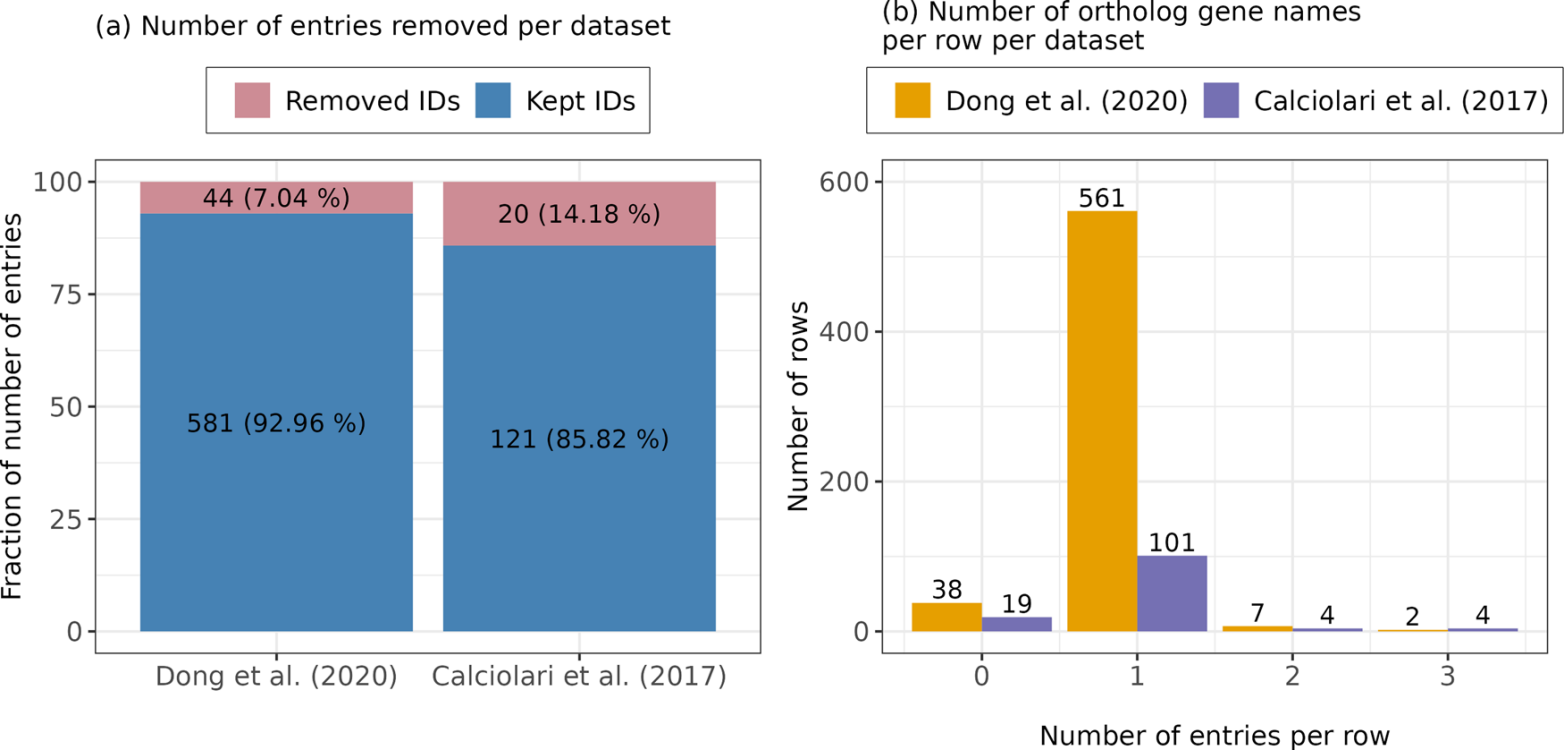
Loss of gene names after ortholog mapping. **a** Assessment of the effectiveness of the “map_orthologs” method on datasets from Dong et al. (2020)^23^ and Calciolari et al. (2017)^22^ that reveals the number of genes that have an ortholog partner in the target organism. **b** Overview of the distribution of ortholog partners in each row for the datasets.

Since gene names differ between organisms, ProHarMeD harmonizes gene names by mapping them to the organism of choice, selected by the user from the list of supported organisms, which currently consists of human, rat, mouse and rabbit. Most studies publish gene lists mapped to human gene names, such that further downstream analysis can be performed, e.g. searching for potential drug targets. Datasets from Schmidt et al. (2016)^20^ and Schmidt et al. (2018)^21^ already contain human genes. The function “map_orthologs” maps gene IDs in the datasets from Calciolari et al. (2017)^22^ and Dong et al. (2020)^23^ from rat and mouse to human orthologs, respectively. Figure 6a shows how many genes in each dataset lack orthologs in human according to the Ensembl database. Even if some individual entries in a row do not have an ortholog partner, this row can be matched to an ortholog if at least one has an ortholog partner. For instance, only 38 rows in the Dong et al. (2020)^23^ dataset remain without a single ortholog gene while 44 genes in the dataset lack a human ortholog partner (Fig. 6b).

### Comparison of study results before and after ID harmonization

**Fig. 7:**
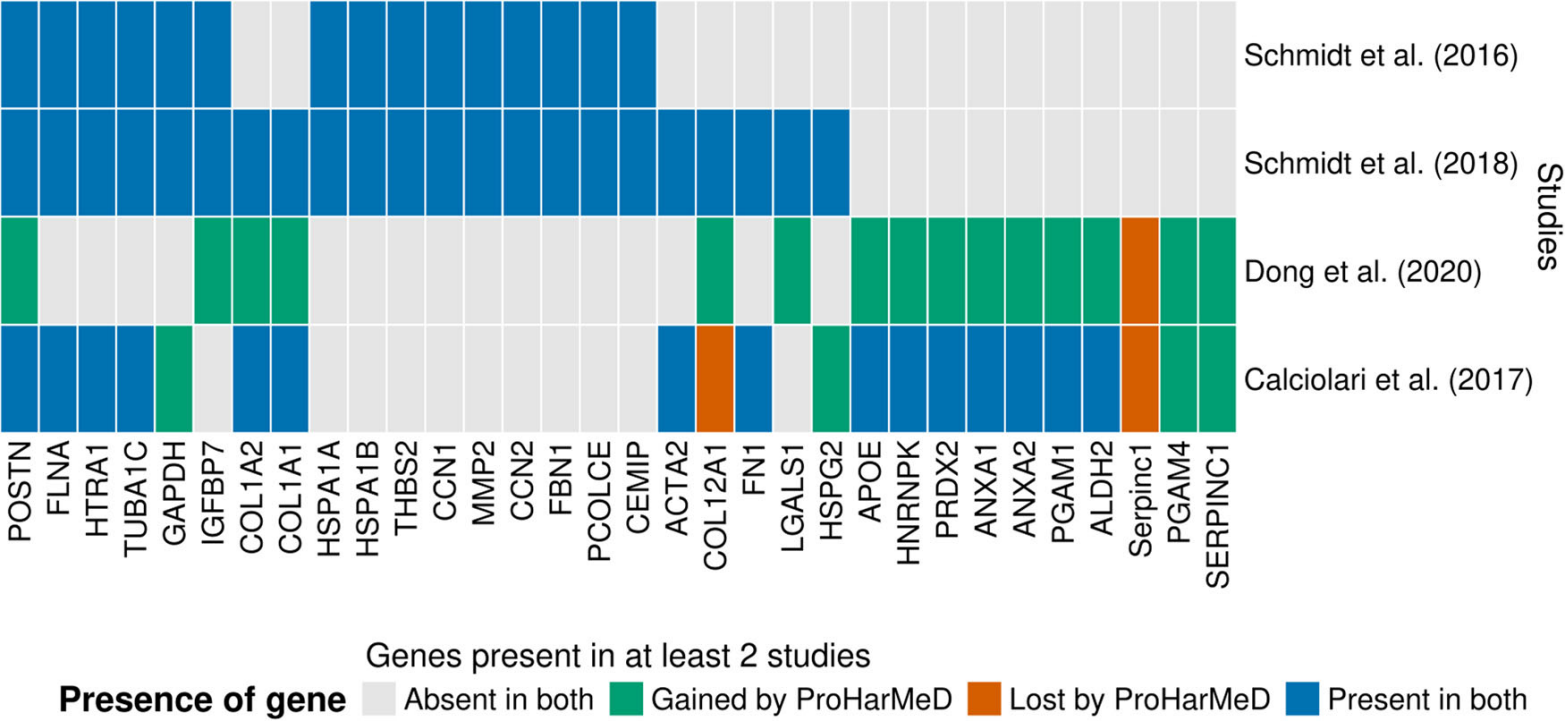
Comparison of the genes present in the published gene lists and the harmonized gene lists with ProHarMeD (see Data Availability Section) using intersection analysis. Only genes identified in at least two studies are displayed.

Upon mapping each study’s IDs to the same gene identifier space, we assess shared IDs, so IDs present in more than one study, with an intersection analysis between the harmonized study results and the published gene lists to demonstrate the benefit of ProHarMeD (Fig. 7). Before harmonization, intersection analysis showed that no gene was present in all four studies (Fig. 7, blue tiles). Moreover, the study by Dong et al. (2020)^23^ has little overlap with the other studies, which is due to the utilization of a murine model and thus, reporting of murine IDs as published gene lists. After harmonization, *POSTN* is found in all four studies. This is a reasonable finding since POSTN’s biological functions for regeneration and wound-healing processes suggest that it is a biomarker for bone healing^26^. Additionally, ProHarMeD greatly increased the overlap of Dong et al. (2020)^23^ study results with the results of other studies due to the mapping of murine genes to human orthologs. This led to a better agreement between studies by Dong et al. (2020)^23^ and Calciolari et al. (2017)^22^, which is expected given that rats and mice are more closely related to one another than humans. Even though a rat osteoporosis model was applied in the study by Calciolari et al. (2017)^22^, most but not all of the genes in the published list have already been mapped to human IDs by the original study’s authors.

The remaining genes still allocated to the rat organism were mapped to orthologous human genes. This introduced four additional human genes (*GAPDH*, *HSPG2*, *PGAM4*, *SERPINC1*) that overlap with the remaining three studies. As previously mentioned, the published gene list of the Calciolari et al. (2017)^22^ dataset contains a few rat genes, leading to the intersection of *Serpinc1* with the published murine gene list of the study by Dong et al. (2020)^23^. After harmonization, this overlap is changed to the human ortholog *SERPINC1*. Lastly, the gene *Col12a1* is missing in the harmonized gene list. This is expected, because the rat proteins *P70560*, *A0A8I6B493*, *A0A8I5ZRE6*, and *A0A0G2KAJ7*, which are encoded by *Col12a1*, are not included in the published protein list of Calciolari et al. (2017)^22^. Unexpectedly, *Col12a1* is listed in the published gene list of this study. Since these proteins are not found in the corresponding published protein list, the raw data from this study would be necessary to verify the validity, which highlights the importance of FAIR. However, Calciolari et al. (2017)^22^ raw mass spectra data is not available.

For our meta-analysis, we only consider proteins that are present in at least two studies. Before harmonization, 21 genes fulfilled this criterion in the “uncleaned” published data sets (see Supplementary Table 1). Thanks to ProHarMeD, the intersection size, which is the number of occurrences of the genes in the four studies, for 5 out of the 21 genes was increased (Fig. 7). More importantly, the intersection analysis after harmonization identified 10 additional genes, representing an increase of about 50%. In one case, the rat gene *Serpinc1* identified before harmonization, was replaced with the human ortholog *SERPINC1* after harmonization, bringing all identified genes to the same organism space. Together, this demonstrates the utility of ProHarMeD, enriching the set of potential biomarkers to 31 genes of interest.

### Identification of drug candidates using meta-study mechanotyping

To perform network-based drug repurposing, we used the 31 biomarkers obtained from our meta-analysis (Fig. 7) as seeds. For seed protein integration, network computation and visualization, we build in the Drugst.One^27^ package, which taps into the NeDRex database^14^, incorporates data from numerous biomedical databases like OMIM^28^, DisGeNET^29^, UniProt^17^, NCBI gene info^30^, IID^31^, MONDO^32^, DrugBank^33^, Reactome^34^, and DrugCentral^35^, and offers a combined protein-drug-disease network. After mapping the 31 seeds to that network, 24 of them spanned a connected subnetwork in the human protein-protein interactome. We then used the “Drug Target Search” option under the “Analysis” section in the ProHarMeD web app, where the Multi-Steiner Trees (MuST)^36^ approach is used to discover connector nodes that are required to connect the seven isolated proteins to the already connected seed nodes. This resulted in the identification of seven connector proteins (see Supplementary Table 2), including *EGFR* and *CTFR*, which are known for impacting bone healing. *EGFR* is an epidermal growth factor receptor that has been found to influence bone formation by negatively inhibiting mTOR signaling during osteoblast differentiation^37^. Cystic fibrosis transmembrane conductance regulator (*CFTR*) mutations affect both osteoblast and osteoclast development^38^. The statistical significance of the resultant network is evaluated using DIGEST^9^, which compares the network to 1000 random networks with the same network attributes regarding functional coherence and calculates an empirical *p*-value. The resultant network outperforms random networks in terms of gene ontology based on biological process (*P*-Value: 0.041), cellular component (*P*-Value: 0.001), and KEGG (*P*-Value: 0.001) (see Supplementary Note 1).

**Fig. 8:**
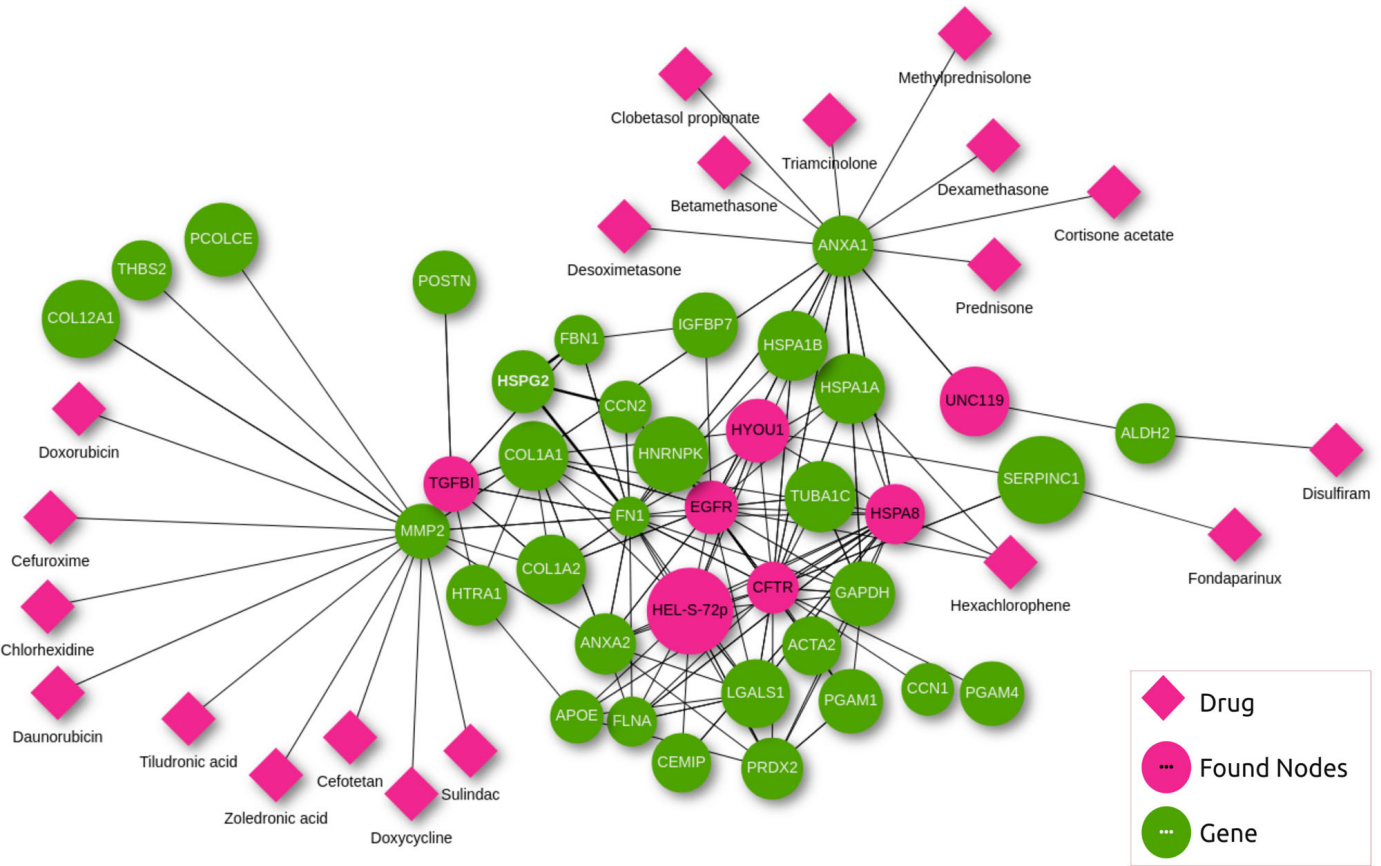
Integrated network visualization of the 31 seeds (green) at the ProHarMeD website. Pink circles are found nodes used to connect the seeds which were identified with MuST^36^. Pink diamonds are the top-20 drugs targeting proteins in the candidate mechanism(s).

Finally, we used the 31 seeds and seven connector proteins to retrieve known drugs using the “Drug search” option under the “Analysis” section of the ProHarMeD web app. The seeds and connector proteins were examined as potential drug targets by retrieving known protein-drug interactions from DrugBank and DrugCentral and visualizing those interactions within the network (Fig. 8). The top-20 identified drugs (see Supplementary Table 3) targeting this network are mostly connected to *MMP2* or *ANXA1*. However, the drug with the highest score was Fondaparinux, which targets the gene *Serpinc1* and has previously been linked to a positive histological effect on bone healing^39^. Nevertheless, the drug target *MMP2* and a number of other identified drug repurposing candidates have been associated with bone regeneration, protection or loss in the literature. The matrix metalloproteinase 2 (*MMP*2) inhibitor 1 (*MMP2-I1*) has a beneficial function in the osteogenesis of human bone marrow mesenchymal stem cells (hBMSCs) by activating the p38/mitogen-activated protein kinase (MAPK) signaling pathway, resulting in increased bone production^40^. The nine drugs connected to *MMP2* include Doxycycline, which is a tetracycline antibiotic that is used to treat various bacterial infections^41^. However, Doxycycline is also known to stimulate bone healing and alter Wnt signaling^42^. Zoledronic acid and Tiludronic acid, both bisphosphonate compounds, were explored separately for their influence on bone regeneration but appear to neither improve nor impair it^43,44^.

Annexin A1 (*ANXA1*) has anti-inflammatory activity^45^ and the identified drugs targeting this gene are steroids used to downregulate inflammation^46–49^. However, a few specific drugs targeting *ANXA1* have been also reported in the context of bone healing. Dexamethasone, a glucocorticoid, aided metaphyseal injury healing, yet long-term glucocorticoid therapy is known to have a deleterious effect on bone growth^50,51^. Some of the identified top-20 drug candidates are known to negatively influence bone healing. Cefuroxime, which is used for the treatment of a variety of infections^52^, was reported to interfere with fracture healing more than a placebo^53^. Doxorubicin, a frequently used chemotherapy treatment, is associated with bone loss^54^. Lastly, the anti-inflammatory drug Sulindac was connected to enhanced osteoblast cell death after hypoxia, leading to a delay of fracture healing in bone tissue under hypoxic injury^55^. The top-20 drugs mentioned here are approved drugs, serving as a starting point for investigating their potential repurposing for bone healing, while ProHarMeD’s flexibility also enables the inclusion of drugs, that are not approved yet, such as those in clinical trials (see Supplementary Table 4).

Of note, our main goal was to present the functionalities of ProHarMeD rather than providing a comprehensive drug-repurposing study for improved bone healing. However, the results identified drugs and drug targets that have previously been studied for their potential in bone healing and regeneration. This confirms the analysis method, which should be utilized on a larger number of studies in the future to guarantee the generalizability of the findings.

In summary, ProHarMeD not only boosted the number of detectable biomarker candidates across several studies by harmonizing the data, but it also facilitated the interactive extraction of disease mechanisms and the selection of drug-repurposing targets and drug candidates. All steps can be performed online at ProHarMeD’s user-friendly web interface for users without any programming experience. In addition, ProHarMeD is available as R and Python packages to allow for pipeline integration. Note that, the datasets used in the example are available on the web interface, allowing the user to recreate the use case. The user can either append the collection of tutorial studies or analyze and integrate any number of datasets of their choice as long as there is a column containing either protein IDs or gene IDs.

### Use case 2: Transcriptomics datasets for meta-analysis

ProHarMeD is not per se restricted to proteomics data; it may also be utilized for other omics data types, most notably transcriptomics data. We exemplify its applicability to gene expression data using two transcriptomics studies^56,57^ (see Supplementary Table 5) on neuroendocrine cancer in human patients. The harmonization procedures are analogous to proteomics pipelines but can be bypassed here, since the two studies acquired data from human patients. Afterwards, similar to proteomics data, all genes are mapped to the molecular interaction networks integrated with NeDRex, and the MuST algorithm was used to generate subnetworks (see Supplementary Table 6). Finally, we used the Drugst.One integration to identify the top five drugs that are linked to the newly found genes (see Supplementary Table 7, Supplementary Fig. 1). Sorafenib, the best-ranked drug, is used to treat hepatocellular carcinoma, advanced renal cell carcinoma, and thyroid carcinoma^58^. Despite being linked to genes that were not previously identified as biomarkers for neuroendocrine carcinoma, Sorafenib is already being investigated as a potential treatment for these malignancies^59^.

## Methods

### Filtering of protein IDs

To verify and possibly remove incorrectly mapped or obsolete protein IDs, ProHarMeD retrieves information from UniProt to obtain the reviewed status and assigned organism. For that, we use the most recent UniProt version, assessed via API. Additionally, this method handles decoy and contaminant IDs (as flagged by MaxQuant), allowing the user to keep or remove them.

The user can choose one or multiple filtering options for the protein IDs:

- **organism-based:** All IDs assigned to other organisms than the given one will be filtered out;
- **reviewed-based**: All IDs that do not have the reviewed status in UniProt will be filtered out;
- **decoy-based**: All IDs that are contaminants (flagged “CON__”) e.g. originating from cell culture medium or mycobacterial contaminations, and decoy proteins (flagged “REV__”), included from target-decoy FDR validation, will be filtered out.

The user also has a choice as to whether the data’s original protein ID column should be replaced or a new column added.

### Re-mapping of gene and protein names

Besides protein IDs, gene names are needed for easier naming in plots and in analytical procedures such as enrichment analysis. In some cases, genes associated with the quantified protein groups in proteomic data are missing.

With direct API access to the UniProt database, ProHarMeD facilitates retrieving the assigned gene names given protein IDs and filling in any missing associations in the data matrix or even replacing ones that already exist, to keep all the names consistent within the same database version.

ProHarMeD implements numerous scenarios in which names can be chosen, including:

- **FASTA**: Use information extracted from FASTA headers, if a user would rather use gene information from their own FASTA file than directly from the UniProt database;
- **UniProt**: Use mapping information from UniProt and use all gene names that are annotated in the HUGO Gene Nomenclature Committee (HGNC)^60^;
- **UniProt_primary**: Use mapping information from UniProt and use only primary gene names;
- **UniProt_one**: Use mapping information from UniProt and use only the most frequent single gene name;
- **All**: Use primarily information extracted from FASTA headers and fill missing entries with data from UniProt.

### Reduction of gene names

Some gene names have multiple synonyms, which creates a potential source of errors when determining intersections between studies, such as undetected overlaps.

Using several attributes and databases, ProHarMeD enables the reduction of the gene names to a single gene name, preventing redundancy.

ProHarMeD offers numerous scenarios for how names can be reduced:

- **Ensembl**: Use the g:Profiler package to reduce gene names to those having an Ensembl ID and use the gene name listed by the Ensembl database;
- **HGNC**: Use the HGNC database^60^ to reduce gene names to those having an entry in HGNC (only for human);
- **MyGeneInfo**: Use the MyGene.info database^18^ to reduce gene names to those having an entry in MyGene.info;
- **Enrichment**: Use the g:Profiler package to reduce gene names to those having a functional annotation.

Note that none of the data repositories is directly integrated with ProHarMeD but queried on the fly such that always the newest release of the respective database is utilized.

### Mapping of orthologs

To perform meta-analysis on studies performed in different organisms, the identifiers of each study must be mapped to the same organism by assigning their orthologous counterpart.

This method converts the gene names of the current organism to the ortholog genes of the target organism using g:Profiler, which uses the information from the Ensembl database. The mapping is carried out in two steps: first, the user-provided input gene IDs are converted to Ensembl gene identifiers, and then the corresponding orthologous gene information for the target organism is retrieved. Both, the original organism and the target organism, must be from the supported list of organisms, here, human, rat, mouse, or rabbit.

The user has the option to retain rows with empty entries resulting from removed or unmappable IDs in all four harmonization functions. Alternatively, they can choose to automatically delete these rows, which is the default behavior.

### Logging

ProHarMeD includes an automated logging function that tracks the success of each of the identifier-changing methods listed, which allows the user to determine the success of conversion.

In addition to returning the input data with altered identifiers, each method call automatically returns logging information separated into two types:

- **Overview Log**: A row-by-row listing of the previous IDs, the altered IDs that remained, the removed IDs, and, if applicable, the added IDs, along with the amount for each;
- **Detailed Log**: A list of the affected identifiers and any additional information from the relevant databases, which may vary depending on the method call but is typically used to better understand the reason for identifier removal. For instance, in the method “Filter_protein_ids” the linkage of the protein ID with the incorrect organism can result in the removal of the ID.

Additionally, ProHarMeD provides built-in visualizations for displaying the logging results.

### Mechanotyping and drug repurposing prediction

With the use of ProHarMeD, the harmonization issue can be resolved, allowing for the comparison of several studies in a meta-study analysis. For this, ProHarMeD enables running an intersection analysis, rating the proteins according to the number of studies they occur in. The user may then select a list of proteins from these results and pipe them as seeds into the mechanism mining pipeline available through the ProHarMeD website.

- **Network Integration**: The proteins are integrated into a network of choice, such as BioGRID^61^, IID^31^, String^62^, APID^63^, IntAct^64^, or into the whole NeDReX network^14^, which combines all of them, in order to study their interconnections.
- **Disease Module Mining**: In cases where not all seeds are directly connected, a Multi-Steiner Tree (MuST) algorithm^36^ can be employed using the “Connect genes” functionality to connect the seeds. We also support other disease module mining tools, available via the “Drug Target Search” task under “Analysis,” e.g., KeyPathwayMiner^65^, TrustRank^66^, or centrality measures such as harmonic, closeness, degree and betweenness centrality.
- **Drug Repurposing Candidate Identification**: The user can finally utilize the centrality measures or TrustRank to identify and rank drugs known to target the proteins in the mechanism displayed as a network and to visualize the results accordingly.

### Implementation

ProHarMeD is available as a Python package (https://pypi.org/project/proharmed/) and an R package (https://github.com/symbod/proharmed-R). Additionally, we offer a website (https://apps.cosy.bio/proharmed) for direct usage for users without programming experience. Using the web tool allows scientists without programming knowledge to conduct all data analyses in one place, create statistical summary plots, and employ the integrated network-based analysis interactively (human in the loop). However, for incorporation into existing pipelines, users and software developers can opt for downloading either our R or our Python ProHarMeD packages. Both offer the same functionalities.

### Reporting summary

Further information on research design is available in the Nature Research Reporting Summary linked to this article.

### Supplementary information

Supplement of the article

Reporting Summary

## Data Availability

The harmonized and not harmonized protein and gene lists^20–23^ utilized in this study are made available through the source code repository (https://github.com/symbod/proharmed). Additionally, all datasets used in this research have been integrated into the website (see section Implementation). The website allows users to reproduce each result step by step without requiring any additional programmatic knowledge.

## Code availability

The Python package source code can be accessed on GitHub at https://github.com/symbod/proharmed or directly from the Python package repository https://pypi.org/project/proharmed/. Furthermore, for those interested in the R implementation, the corresponding R code is also available at https://github.com/symbod/proharmed-R.
