# Supplementary material for: Proteomic meta-study harmonization, mechanotyping and drug repurposing candidate prediction with ProHarMeD
Source: NPJ Syst Biol Appl. 2023 Oct 10;9:49. doi: 10.1038/s41540-023-00311-7 (PMC10564802; doi:10.1038/s41540-023-00311-7)
Supplement: Supplementary file 1 — Supplement of the article [file 41540_2023_311_MOESM1_ESM.pdf]

Supplementary Information of the Article:

Proteomic Meta-Study Harmonization, Mechanotyping and Drug Repurposing Candidate Prediction with ProHarMeD

Klaudia Adamowicz [1,*], Lis Arend [1], Andreas Maier [1], Johannes R. Schmidt [2], Bernhard Kuster [3], Olga Tsoy [1], Olga Zolotareva [1,4], Jan Baumbach [1,5], Tanja Laske [1]

[1]Institute for Computational Systems Biology, University of Hamburg, Hamburg, 22607, Germany
[2]Department of Preclinical Development and Validation, Fraunhofer Institute for Cell Therapy and Immunology IZI, Leipzig, Germany
[3]Chair of Proteomics and Bioanalytics, Technical University of Munich, Freising, Germany
[4]Chair of Experimental Bioinformatics, TUM School of Life Sciences, Technical University of Munich, Freising, Germany
[5]Department of Mathematics and Computer Science, University of Southern Denmark, Odense, 5230, Denmark

Supplementary Notes

Supplementary Note 1. Result for the significance of final network based on functional coherence.
https://digest-validation.net/result?id=8a2284a7-8022-455c-9bd6-73d8b0ab858f

Supplementary Tables

Supplementary Table 1. Identified genes through intersection analysis of the non-harmonized published gene list.

symbol	uniprot	ensg	entrez	proteinName
FN1	B7ZLE5|P02751	ENSG00000115414	2335	fibronectin
COL12A1	Q99715	ENSG00000111799	1303	collagen alpha-1(XII) chain
THBS2	P35442	ENSG00000186340	7058	thrombospondin-2
PCOLCE	Q15113	ENSG00000106333	5118	procollagen C-endopeptidase enhancer 1
FBN1	P35555	ENSG00000166147	2200	fibrillin-1
FLNA	Q60FE5|P21333|Q6NXF2	ENSG00000196924	2316	filamin-A
GAPDH	P04406	ENSG00000111640	2597	glyceraldehyde-3-phosphate dehydrogenase

TUBA1C	Q9BQE3|F5H5D3	ENSG00000167553	84790	tubulin alpha-1C chain
HSPA1A	P0DMV8	undefined	3303	heat shock 70 kDa protein 1A
HSPA1B	P0DMV9	undefined	3303	heat shock 70 kDa protein 1A
CCN1	O00622	ENSG00000142871	3491	CCN family member 1
ACTA2	D2JYH4|P62736	ENSG00000107796	59	actin, aortic smooth muscle
CCN2	P29279	ENSG00000118523	1490	CCN family member 2
COL1A1	P02452	ENSG00000108821	1277	collagen alpha-1(I) chain
COL1A2	P08123|A0A0S2Z3H5	ENSG00000164692	1278	collagen alpha-2(I) chain
POSTN	Q15063|B1ALD9|A0A024RDT5|A0A024RDS2	ENSG00000133110	10631	periostin
IGFBP7	Q16270	ENSG00000163453	3490	insulin-like growth factor-binding protein 7
SERPINC1	A0A024R944|P01008	ENSG00000117601	462	antithrombin-III
HTRA1	Q92743	ENSG00000166033	5654	serine protease HTRA1
MMP2	P08253|A0A024R6R4	ENSG00000087245	4313	72 kDa type IV collagenase
CEMIP	Q8WUJ3	ENSG00000103888	57214	cell migration-inducing and hyaluronan-binding protein

Supplementary Table 2. Gene list of the final network. The boolean in the column isSeed indicates if it was an input seed gene or a found gene to connect the seed genes.

symbol	uniprot	ensg	entrez	proteinName	isSeed
FN1	B7ZLE5|P02751	ENSG00000115414	2335	fibronectin	true
ALDH2	P05091|A0A384NPN7	ENSG00000111275	217	aldehyde dehydrogenase, mitochondrial	true
COL12A1	Q99715	ENSG00000111799	1303	collagen alpha-1(XII) chain	true
THBS2	P35442	ENSG00000186340	7058	thrombospondin-2	true
PCOLCE	Q15113	ENSG00000106333	5118	procollagen C-endopeptidase enhancer 1	true
FBN1	P35555	ENSG00000166147	2200	fibrillin-1	true
FLNA	Q60FE5|P21333|Q6NXF2	ENSG00000196924	2316	filamin-A	true
PRDX2	P32119	ENSG00000167815	7001	peroxiredoxin-2	true
GAPDH	P04406	ENSG00000111640	2597	glyceraldehyde-3-phosphate dehydrogenase	true
HYOU1	A0A494C039|Q6IN67|Q9Y4L1	ENSG00000280682|ENSG00000149428	10525	hypoxia up-regulated protein 1	false
TUBA1C	Q9BQE3|F5H5D3	ENSG00000167553	84790	tubulin alpha-1C chain	true
HSPA1A	P0DMV8	undefined	3303	heat shock 70 kDa protein 1A	true
HSPA1B	P0DMV9	undefined	3303	heat shock 70 kDa protein 1A	true
UNC119	Q13432|K7EN86	ENSG00000109103	9094	protein unc-119 homolog A	false
EGFR	E7BSV0|C9JYS6|E9PFD7|P00533|Q504U8	ENSG00000146648	1956	epidermal growth factor receptor	false
CCN1	O00622	ENSG00000142871	3491	CCN family member 1	true

ACTA2	D2JYH4|P62736	ENSG00000107796	59	actin, aortic smooth muscle	true
CCN2	P29279	ENSG00000118523	1490	CCN family member 2	true
HSPA8	P11142	ENSG00000109971	3312	heat shock cognate 71 kDa protein	false
COL1A1	P02452	ENSG00000108821	1277	collagen alpha-1(I) chain	true
APOE	P02649|A0A0S2Z3D5	ENSG00000130203	348	apolipoprotein E	true
COL1A2	P08123|A0A0S2Z3H5	ENSG00000164692	1278	collagen alpha-2(I) chain	true
POSTN	Q15063|B1ALD9|A0A024RDT5|A0A024RDS2	ENSG00000133110	10631	periostin	true
HEL-S-72p	V9HW22	ENSG00000109971	3312	heat shock cognate 71 kDa protein	false
IGFBP7	Q16270	ENSG00000163453	3490	insulin-like growth factor-binding protein 7	true
SERPINC1	A0A024R944|P01008	ENSG00000117601	462	antithrombin-III	true
HTRA1	Q92743	ENSG00000166033	5654	serine protease HTRA1	true
PGAM1	P18669|Q6FHU2	ENSG00000171314	5223	phosphoglycerate mutase 1	true
PGAM4	Q8N0Y7	ENSG00000226784	441531	phosphoglycerate mutase 4	true
ANXA1	Q5TZZ9|P04083	ENSG00000135046	301	annexin A1	true
ANXA2	P07355|A0A024R5Z7|H0YL33	ENSG00000182718	302	annexin A2	true
HNRNPK	P61978	ENSG00000165119	3190	heterogeneous nuclear ribonucleoprotein K	true
MMP2	P08253|A0A024R6R4	ENSG00000087245	4313	72 kDa type IV collagenase	true

HSPG2	P98160	ENSG00000142798	3339	basement membrane-specific heparan sulfate proteoglycan core protein	true
CEMIP	Q8WUJ3	ENSG00000103888	57214	cell migration-inducing and hyaluronan-binding protein	true
CFTR	A0A024R730|P13569	ENSG00000001626	1080	cystic fibrosis transmembrane conductance regulator	false
LGALS1	P09382	ENSG00000100097	3956	galectin-1	true
TGFBI	Q15582|A0A0S2Z4Q2	ENSG00000120708	7045	transforming growth factor-beta-induced protein ig-h3	false

Supplementary Table 3. Drug list of the final network. Only approved drugs were taken into consideration.

drugId	label	score	rank	status
DB00569	Fondaparinux	1	1	approved
DB00605	Sulindac	0.738916506295296	2	approved
DB00997	Doxorubicin	0.738916506295296	2	approved
DB00878	Chlorhexidine	0.738916506295296	2	approved
DB00254	Doxycycline	0.738916506295296	2	approved
DB01330	Cefotetan	0.738916506295296	2	approved
DB00399	Zoledronic acid	0.738916506295296	2	approved
DB01112	Cefuroxime	0.738916506295296	2	approved
DB01133	Tiludronic acid	0.738916506295296	2	approved
DB00694	Daunorubicin	0.738916506295296	2	approved
DB00822	Disulfiram	0.544866264201855	3	approved
DB00756	Hexachlorophene	0.259380395752685	4	approved
DB01013	Clobetasol propionate	0.154761311119543	5	approved
DB00959	Methylprednisolone	0.154761311119543	5	approved
DB00620	Triamcinolone	0.154761311119543	5	approved
DB00635	Prednisone	0.154761311119543	5	approved
DB01234	Dexamethasone	0.154761311119543	5	approved
DB01380	Cortisone acetate	0.154761311119543	5	approved
DB00547	Desoximetasone	0.154761311119543	5	approved
DB00443	Betamethasone	0.154761311119543	5	approved

Supplementary Table 4. Drug list of the final network. Not (yet) approved drugs were additionally taken into consideration.

drugId	label	score	rank	status
DB01109	Heparin	1	1	unapproved
DB01225	Enoxaparin	1	1	unapproved
DB00569	Fondaparinux	1	1	approved
DB00605	Sulindac	0.738916506295296	2	approved
DB00997	Doxorubicin	0.738916506295296	2	approved
DB00878	Chlorhexidine	0.738916506295296	2	approved
DB11145	Oxyquinoline	0.738916506295296	2	unapproved
DB00254	Doxycycline	0.738916506295296	2	approved
DB01330	Cefotetan	0.738916506295296	2	approved
DB00399	Zoledronic acid	0.738916506295296	2	approved
DB01112	Cefuroxime	0.738916506295296	2	approved
DB03424	Ubenimex	0.738916506295296	2	unapproved
DB04216	Quercetin	0.738916506295296	2	unapproved
DB11617	Aclarubicin	0.738916506295296	2	unapproved
DB01133	Tiludronic acid	0.738916506295296	2	approved
DB00694	Daunorubicin	0.738916506295296	2	approved
DB00822	Disulfiram	0.544866264201855	3	approved
DB00756	Hexachlorophene	0.259380395752685	4	approved
DB00171	ATP	0.259380395752685	4	unapproved
DB01013	Clobetasol propionate	0.154761311119543	5	approved

Supplementary Table 5. Transcriptomic biomarkers for neuroendocrine cancers as gene symbols.

**Study**	**Organism**	**Genes**
Zhang *et al.* (2022) [1]	Human	CHGA, COL2A1, GABRG2, MATN4, NEUROG2, SYT4, SLC6A17
Balanis *et al.* (2019) [2]	Human	CHGA, SYP, NCAM1, TTF1

Supplementary Table 6. Gene list of the final network for neuroendocrine cancers. The boolean in the column isSeed indicates if it was an input seed gene or a found gene to connect the seed genes.

**symbol**	**entrez**	**proteinName**	**isSeed**
SLC6A17	388662	sodium-dependent neutral amino acid transporter SLC6A17	true
CHGA	1113	chromogranin-A	true
MATN4	8785	matrilin-4	true
SYP	6855	synaptophysin	true
NEUROG2	63973	neurogenin-2	true
TTF1	7270	transcription termination factor 1	true
NCAM1	4684	neural cell adhesion molecule 1	true
SYT4	6860	synaptotagmin-4	true
COL2A1	1280	collagen alpha-1(II) chain	true
GABRG2	2566	gamma-aminobutyric acid receptor subunit gamma-2	true
NTRK1	4914	high affinity nerve growth factor receptor	false
PARK2	5071	E3 ubiquitin-protein ligase parkin	false
MYC	4609	myc proto-oncogene protein	false
HAX1	10456	HCLS1-associated protein X-1	false
SMARCA4	6597	transcription activator BRG1	false
RAF1	5894	RAF proto-oncogene serine/threonine-protein kinase	false
BSCL2	26580	seipin	false

KRAS	3845	GTPase KRas	false
LAMP2	3920	lysosome-associated membrane glycoprotein 2	false

Supplementary Table 7. Identified drugs targeting newly found nodes from supplementary table 6.

**label**	**drugId**	**score**	**rank**	**status**
Sorafenib	DB00398	1	1	approved
Encorafenib	DB11718	0.54877863047828	2	approved
Nilotinib	DB04868	0.54877863047828	2	approved
Dasatinib	DB01254	0.54877863047828	2	approved
Vemurafenib	DB08881	0.54877863047828	2	approved

Supplementary Figures

Supplementary Figure 1: Proposed neuroendocrine cancer-specific subnetwork including the top 5 scored drugs connected to the new found nodes.

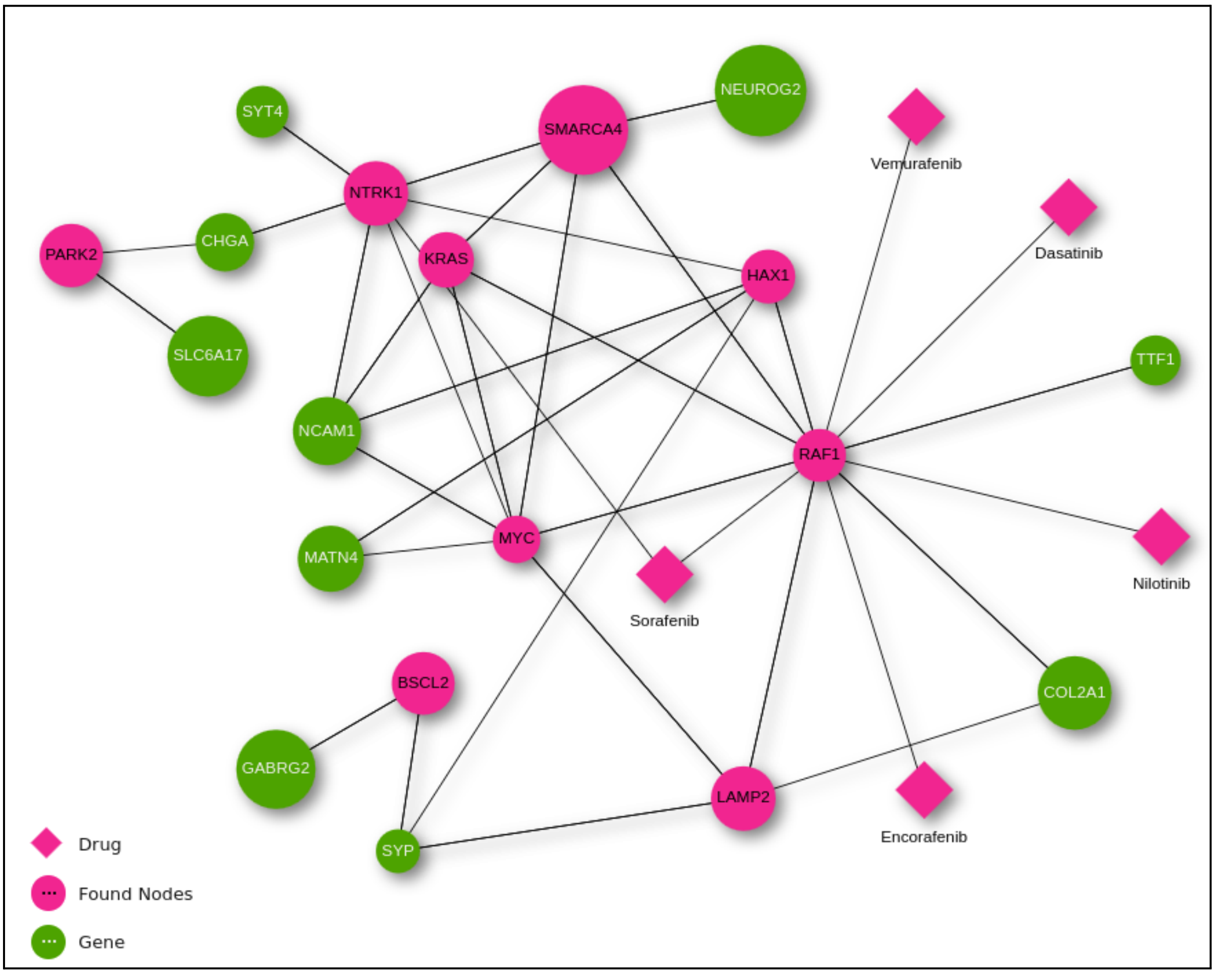

Supplementary References

1. Zhang, Y., Yang, L. & Jiao, X. Analysis of Breast Cancer Differences between China and Western Countries Based on Radiogenomics. *Genes* **13**, 2416 (2022).
2. Balanis, N. G. *et al.* Pan-cancer convergence to a small cell neuroendocrine phenotype that shares susceptibilities with hematological malignancies. *Cancer Cell* **36**, 17-34.e7 (2019).
